# Improving focality and consistency in micromagnetic stimulation

**DOI:** 10.3389/fncom.2023.1105505

**Published:** 2023-02-02

**Authors:** Hui Ye, Vincent Hall, Jenna Hendee

**Affiliations:** Department of Biology, Loyola University Chicago, Chicago, IL, United States

**Keywords:** micromagnetic stimulation (μMS), figure-eight micro-coil, activating function, NEURON modeling, ion channels, consistency

## Abstract

The novel micromagnetic stimulation (μMS) technology aims to provide high resolution on neuronal targets. However, consistency of neural activation could be compromised by a lack of surgical accuracy, biological variation, and human errors in operation. We have recently modeled the activation of an unmyelinated axon by a circular micro-coil. Although the coil could activate the axon, its performance sometimes lacked focality and consistency. The site of axonal activation could shift by several experimental factors, including the reversal of the coil current, displacement of the coil, and changes in the intensity of the stimulation. Current clinical practice with transcranial magnetic stimulation (TMS) has suggested that figure-eight coils could provide better performance in magnetic stimulation than circular coils. Here, we estimate the performance of μMS by a figure-eight micro-coil, by exploring the impact of the same experimental factors on its focality and consistency in axonal activation. We derived the analytical expression of the electric field and activating function generated by the figure-eight micro-coil, and estimated the location of axonal activation. Using NEURON modeling of an unmyelinated axon, we found two different types (A and B) of axon activation by the figure-eight micro-coil, mediated by coil currents of reversed direction. Type A activation is triggered by membrane hyperpolarization followed by depolarization; Type B activation is triggered by direct membrane depolarization. Consequently, the two types of stimulation are governed by distinct ion channel mechanisms. In comparison to the circular micro-coil, the figure-eight micro-coil requires significantly less current for axonal activation. Under figure-eight micro-coil stimulation, the site of axonal activation does not change with the reversal of the coil current, displacement of the coil, or changes in the intensity of the stimulation. Ultimately, the figure-eight micro-coil provides a more efficient and consistent site of activation than the circular micro-coil in μMS.

## 1. Introduction

Transcranial magnetic stimulation (TMS) is a noninvasive brain stimulation technique used in the clinic to treat several neurological disorders and psychiatric diseases. One of TMS’s major limitations is its low spatial resolution, which often results in a mismatch between the target area in the brain and the stimulation site on the scalp. Early TMS practices using a single circular coil were soon found to experience difficulties in locally stimulating the targeted areas in the brain (Barker et al., 1985). It took only a few years before the figure-eight coil was developed for more localized brain stimulation (Ueno et al., 1988). Using idealized coils consisting of one or two wire loops, it was soon demonstrated that figure-eight coils allow for more focused stimulation than simple round coils (Ravazzani et al., 1996). In recent years, different variants of the figure-eight coils have been developed for basic research and clinical purposes (Ueno and Sekino, 2021), such as the quadruple butterfly coil (Rastogi et al., 2017), eccentric figure-eight coil (Sekino et al., 2015), and figure-eight coil with an iron core (Yamamoto et al., 2016), etc. Applying these novel designs to neural stimulation has led to an in-depth understanding of the functional organization of the human brain, dynamic neuronal connectivity, and neuronal plasticity in the cortex.

An effective way to increase the spatial resolution of magnetic stimulation is by reducing the dimensionality of the stimulating coil (Talebinejad and Musallam, 2010; Tischler et al., 2011). This led to the recent development of micromagnetic stimulation (μMS) technology, which significantly increases the focality of magnetic stimulation. This technology uses millimeter or submillimeter coils to target individual neurons (Bonmassar et al., 2012; Ye and Barrett, 2021) or specific neuronal populations (Lee and Fried, 2017). Due to their microscopic size, these coils are meant to be implanted close to the targeted neurons or axons under the cover of biocompatible materials (Park et al., 2013). This prevents direct contact between the coil and the neural tissue, mitigating numerous adverse effects that arise at the brain-electrode interface when metal electrodes are implanted for neural stimulation (Polikov et al., 2005; Cogan, 2008; Koivuniemi et al., 2011). Several works have used μMS in axonal activation, including the axons of the hippocampus (Saha et al., 2022a), apical dendrites of layer V pyramidal neurons in the cortex (Lee and Fried, 2017), and axons in the dorsal cochlear nucleus (Golestanirad et al., 2018).

The ultimate goal of the novel μMS technology is to achieve highly specific neural stimulation with improved consistency, defined by a micro-coil’s ability to repetitively generate the same neural response. However, there is a considerable chance that μMS could generate inconsistent results, introduced by a lack of surgical accuracy, multiple biological variations, and human errors in operation.

The permanent and irreversible implantation surgery could cause inconsistency in μMS. Because of its small dimension, it is difficult to implant the micro-coil at the same anatomical location across different subjects. Recent technological developments in surgical robotics can mitigate the effects of human hand tremors within the micrometer scale (Ahmed et al., 2018). However, placement of implants is still at the millimeter (mm) level of precision (Fernandes de Oliveira Santos et al., 2021; Zhang et al., 2021). Therefore, even with assistance from surgical robots and software, it is difficult to achieve implantation that is precise to the micrometer (μm) scale. It is highly important to ensure the stimulation apparatus (i.e., a micro-coil) is as focal and consistent as possible during μMS.

Variance in the biological system could cause inconsistency in μMS. First, neural activation is determined by the direction, intensity, and duration of the electric field and its spatial derivative along the neural tissue (Pashut et al., 2014). Consequently, the orientation of the targeted neurons to the micro-coil is crucial for neural activation (Golestanirad et al., 2018). Yet, ensuring the implanted coil has the ideal orientation to the targeted tissue is challenging. Second, most clinical purposes require a high degree of accuracy and precision when small neuronal volumes are targeted for treatment. However, numerous factors could cause migration of the implanted micro-device (van den Munckhof et al., 2010; Park et al., 2011), such as pulsing of blood in the vessels, breathing, or simple locomotion. Device migration would alter the neuronal population being activated.

Human errors in operating the stimulation protocols could cause inconsistency in μMS. Accidentally choosing the wrong intensity and polarity for stimulation could lead to inconsistent or unwanted outcomes. In TMS practice, the stimulation outcome is routinely monitored with fMRI or EEG recording (Bohning et al., 1999; Habibollahi Saatlou et al., 2018). This is not an option in μMS, for which the stimulation site is microscopic and only a small population of neurons are affected by the micro-coil. After the micro-coil is chronically implanted, it is difficult to detect the consequence of these human errors by acquiring feedback at the single cell level. Understanding these variabilities and their respective impacts on stimulation outcomes could significantly improve the development of μMS technology and maximize the device’s success.

To further increase the focality of μMS and the directionality of the neural response, researchers in the μMS field have started to explore the possibility of combining multiple coils to increase stimulation effectiveness. Jeong et al. (2021) designed a figure-eight spiral micro-coil to generate high magnetic flux for ultra-focal stimulation. Saito constructed a figure-eight micro-coil using commercially available chip inductors, which effectively suppressed synchronized bursting activity in a cultured neural network (Saito, 2021). Colella et al. (2021) simulated axonal stimulation of peripheral nerves with the figure-eight micro-coil, and found that the threshold for axonal activation was lower for the figure-eight coil than for a circular micro-coil.

Computational works have provided invaluable insights to estimate the focality of micro-coil stimulation. Researchers have estimated the induced electric field distribution around the single coil (Bonmassar et al., 2012; Osanai et al., 2018; Ye et al., 2020) and multi-coil systems (Minusa et al., 2019). However, when the strength of the field induced by the micro-coil is small, the coil can still produce a spatial gradient that can activate the neuron (Lee et al., 2022). This field gradient, or activating function, is directly related to the site of neural activation (Rattay, 1986; Lee and Fried, 2017). Therefore, several research endeavors have computed the activating function for the single coil system to identify the site of neural activation (Lee et al., 2016; Ye, 2022). We recently simulated axonal activation by a circular micro-coil using activating function analysis and NEURON simulation (Ye, 2022). This work suggests that μMS stimulation with a circular micro-coil could be inconsistent, and that the location of axonal activation could shift by several experimental factors, including the reversal of the coil current, increases in the coil-axon distance, and changes in the intensity of stimulation. When a micro-coil with a dimension of 1 mm was tested, the activation site on the axon could shift by several hundred micrometers (Ye, 2022). So far, activating function analysis has not been performed to estimate the focality and consistency of stimulation in a figure-eight coil system for the activation of a straight axon. Furthermore, although it was believed that the figure-eight micro-coil could provide a better “coupling between nerve fibers and the induced electric field” (Colella et al., 2021), there is a lack of understanding of the neuronal mechanisms underlying axonal activation by such a coil.

This paper will examine the consistency of axonal activation by a figure-eight micro-coil, and investigate the underlying ion channel mechanisms using combined activating function analysis and NEURON modeling. We will compare these results from our recent analysis on a single, circular micro-coil (Ye, 2022). This work provides valuable insights to the further development of μMS technology for focal and consistent neural stimulation.

## 2. Materials and methods

### 2.1. Axon model under magnetic stimulation in a figure-eight coil system

**FIGURE 1:**
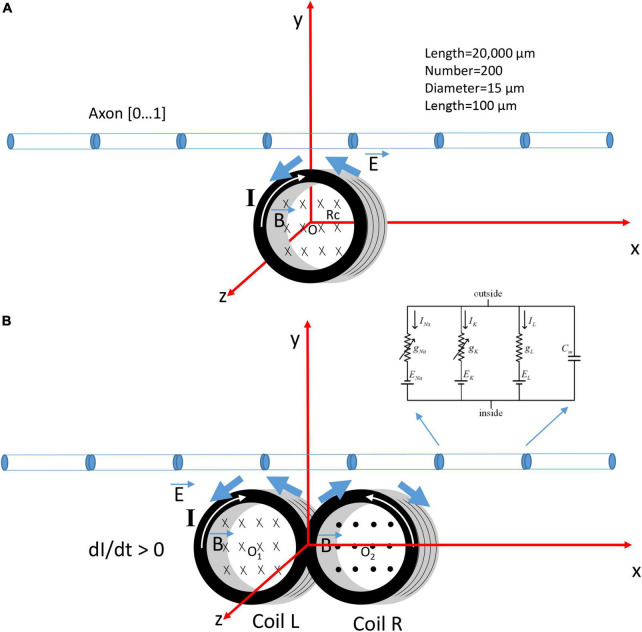
Coordination system that describes the orientation of a circular micro-coil versus a figure-eight micro-coil for the activation of an unmyelinated axon. The coil current (*I*) generated a time-varying magnetic field ($\vec{B}$), which, in turn, induced an electric field ($\vec{E}$) to stimulate the axon. The multi-compartment model of the unmyelinated axon was 20,000 μm in length and was divided into 200 segments. Each segment was a cylinder of length 100 μm and diameter 15 μm. Hodgkin-Huxley type ion channels were inserted into each segment. **(A)** The circular micro-coil had a radius *Rc*. The center of the coil was at O (0,0,0) in the Cartesian coordinate system. The axon was in the x-y plan and was parallel to the x-axis. Increase in the coil current (*I, dI/dt* > 0) in the clockwise direction generated a counterclockwise electric field and a field gradient along the axon. **(B)** The figure-eight micro-coil was made with two identical, circular micro-coils (L and R) located next to each other. The center of the left coil was O_1_ (*–Rc*, 0, 0) and the center for the right coil was O_2_ (*Rc*, 0, 0). Coil current (I) was clockwise in the L-coil, and counterclockwise in the R-coil. The current was increasing with time (*dI/dt* > 0).

Previously, we (Ye, 2022) have modeled a submillimeter, circular micro-coil with radius *Rc* (Figure 1A). To model a figure-eight micro-coil, we positioned two identical micro-coils next to each other, but with reversed winding direction (Figure 1B). The center of the left coil was at point O_1_ (*−Rc*, 0, 0), and the center of the right coil was at point O_2_ (*Rc*, 0, 0). Electric currents in the two coils were synchronized, but with reversed directions. This figure-eight micro-coil was positioned so that its induced electric field was in parallel with the axon, to generate effective stimulation (Jefferys, 1981; Gluckman et al., 1996).

The unmyelinated axon was represented with a multi-compartment model in NEURON (v.7.8) simulation environment package (Hines and Carnevale, 1997). The model simulated the axon as a cylinder 20,000 μm in length and 15 μm in diameter. The axon was divided evenly into 200 node segments [Table 1 in Ye (2022)]. Each segment contained the Hodgkin-Huxley (H-H) types of ion channels, including the fast sodium, slow potassium, and leakage channels in the membrane nodes (Hodgkin and Huxley, 1952). The ionic current (I) at the n-th segment of the neuron was described as,


(1) $$I_{n}=g_{Na}m^{3}h\left(V_{n}-V_{Na}\right)+g_{k}n^{4}\left(V_{n}-V_{k}\right)+g_{L}\left(V_{n}-V_{L}\right)$$

where V_*Na*_, V_*K*_, and V_*L*_ were the equilibrium membrane potentials for sodium, potassium, and leakage channels, respectively. g_*Na*_, g_*k*_, and g_*L*_ were the maximal conductance of sodium, potassium, and leakage channels, respectively. *m* and *h* represented the activation and inactivation of the sodium channels, respectively, whereas *n* represented the activation of potassium channels. The state variables *m, h*, and *n* were defined in,


(2) $$\frac{dm}{dt}=\alpha_{m}\left(1-m\right)-\beta_{m}m$$

(3) $$\frac{dh}{dt}=\alpha_{h}\left(1-h\right)-\beta_{h}h$$

(4) $$\frac{dn}{dt}=\alpha_{n}\left(1-n\right)-\beta_{n}n$$

where α_*m*_, β_*m*_, α_*h*_, β_*h*_, α_*n*_, and β_*n*_ were rate constants defined previously [Table 2 in Ye (2022)].

### 2.2. Electric field induced by a figure-eight micro-coil

Previously, we (Ye, 2022) have derived the analytical expression of the induced electric field generated by a circular micro-coil (Figure 1A). The electric field generated by the figure-eight micro-coil is, therefore, the summation of the two individual circular micro-coils (Left coil and Right coil, Figure 1B). We applied a single voltage pulse (positive or negative) to the figure-eight coil, since this pulse was sufficient to activate various axons (Bonmassar et al., 2012; Lee et al., 2016). Electric fields generated by the left coil (EL) and the right coil (ER) were calculated as follows:

For the onset of the positive stimulation pulse, the electric field generated by the left coil was calculated with Eqs. (13, 14) in Ye (2022),


(5-1) ELx=-Vμ0NRc22Lly(x+Rc)2+y2e-tRL

(5-2) ELy=Vμ0NRc22Llx+Rc(x+Rc)2+y2e-tRL

Electric field generated by the right coil was,


(5-3) ERx=Vμ0NRc22Lly(x-Rc)2+y2e-tRL

(5-4) ERy=-Vμ0NRc22Llx-Rc(x-Rc)2+y2e-tRL

where *N* is the number of coil loops, *l* the coil length, *R* the coil resistance, and L the coil inductance. μ_0_ is the vacuum permeability and *V* is the voltage across the coil. *x* and *y* defined the location considered in the Cartesian coordinate system (Figure 1B), and *t* defined time.

The summarized electric field generated by the figure-eight coil at the onset of a positive stimulation pulse was:


(6-1) Ex=ELx+ERx=Vμ0NRc22Ll

$$\left[\frac{y}{{\left(x-Rc\right)}^{2}+y^{2}}-\frac{y}{{\left(x+Rc\right)}^{2}+y^{2}}\right]e^{-\frac{tR}{L}}$$

(6-2) Ey=ELy+ERy=Vμ0NRc22Ll

$$\left[\frac{x+Rc}{{\left(x+Rc\right)}^{2}+y^{2}}-\frac{x-Rc}{{\left(x-Rc\right)}^{2}+y^{2}}\right]e^{-\frac{tR}{L}}$$

The intensity of the electric field was calculated with,


(7) $$E={\left(Ex^{2}+Ey^{2}\right)}^{1/2}$$

For the offset of the stimulation pulse, the electric field generated by the left coil was calculated by Eqs. (15, 16) from Ye (2022).


(8-1) ELx=Vμ0NRc22Lly(x+Rc)2+y2e-tRL

(8-2) ELy=-Vμ0NRc22Llx+Rc(x+Rc)2+y2e-tRL

Electric field generated by the right coil was,


(8-3) ERx=-Vμ0NRc22Lly(x-Rc)2+y2e-tRL

(8-4) ERy=Vμ0NRc22Llx-Rc(x-Rc)2+y2e-tRL

The summarized electric field generated by the figure-eight micro-coil at the offset of the stimulation pulse was:


(9-1) Ex=ELx+ERx=Vμ0NRc22Ll

$$\left[\frac{y}{{\left(x+Rc\right)}^{2}+y^{2}}-\frac{y}{{\left(x-Rc\right)}^{2}+y^{2}}\right]e^{-\frac{tR}{L}}$$

(9-2) Ey=ELy+ERy=Vμ0NRc22Ll

$$\left[\frac{x-Rc}{{\left(x-Rc\right)}^{2}+y^{2}}-\frac{x+Rc}{{\left(x+Rc\right)}^{2}+y^{2}}\right]e^{-\frac{tR}{L}}$$

Here, *L/R* defined the time constant. Therefore, the single pulse in the figure-eight micro-coil induced a biphasic electric field around the axon. Axon stimulation should occur during the onset phase and/or the offset phase of the stimulus pulse that effectively generates the electric field.

### 2.3. Measuring the induced electric field generated by the figure-eight micro-coil

To validate the mathematic derivation that the magnetically induced electric field by the figure-eight coil is indeed biphasic, we assembled a figure-eight micro-coil (Supplementary Figure 1) using two commercial submillimeter, multilayer surface mount inductors (100 nH, 2 Ω, MLG1005SR10JTD25, TDK USA. Corporation, Uniondale, NY, USA). The internal structure of the coil was visualized by removing the ceramic core and epoxy coating using 40% liquid hydrofluoric acid and 10 N HCL, based on a published protocol (Ye et al., 2020). The coil contained 20 wire loops (*N* = 20).

Two copper wires (magnetic wire 32-AWG, GC electronics, IL, L3-616) were inserted into the shaft of a 20 AWG needle and the barrel of a syringe. They were soldered to the metal leads of the two inductors for electric current delivery. The two inductors were positioned side by side but with reversed orientation to ensure opposite winding of the coil. To ensure electrical insulation and water impermeability of the exposed coil terminals during electrophysiological experiments, the coil was then coated with acrylate copolymer enamel (Park et al., 2013) and allowed 24 h to dry before the experiment. The other end of the copper wires were connected to the pin-connectors. We delivered electric pulses to the figure-eight micro-coil using an arbitrary function generator (AFG1022, Tektronix) and a power amplifier (Pyramid PB 717X 2 channel, Pyramid Car Audio, Brooklyn, NY, 11204, USA). To measure the induced electric field around the figure-eight micro-coil, we filled a petri dish with conductive saline and submerged the figure-eight micro-coil under the saline. We positioned a glass electrode next to the figure-eight micro-coil. The recorded waveform was amplified by a model 1,700 differential AC amplifier (A-M Systems) and stored on a computer with Spike 2 software (v. 7.2 Cambridge Electronic Design Limited).

**FIGURE 2:**
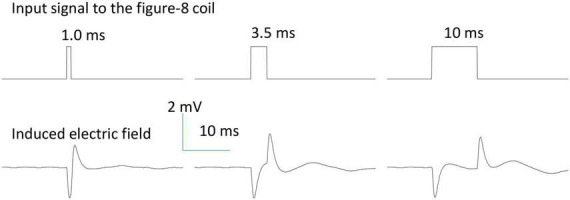
Waveform of the induced electric field generated by a figure-eight micro-coil. Positive voltage pulses (0.2 V) of various durations (1.0, 3.5, and 10 ms) were used to drive the figure-eight micro-coil. The biphasic electric field was generated at the onset and offset phases of the pulse due to the quick change in the coil current.

The assembled figure-eight coils were tested to ensure there was no leakage or current. When the coil was immersed in saline solution, we measured the impedance between one end of the coil to the ground, and found it was greater than 10 MΩ, suggesting a good insulation by the cover material [5 MΩ in Bonmassar et al. (2012)]. In addition, we applied a long pulse (150 ms) to the coil and measured the electric voltage in the petri dish. If the coil was perfectly covered and there was no leaking current, we recorded a quick and large voltage change at the onset and offset of the pulse (Figure 2). If leakage occurred and the terminal end was exposed to the saline, we recorded a much greater voltage at the onset and offset of the pulse. We also recorded a large, non-zero voltage during the whole stimulus pulse period, due to the leakage current. This method provides an easy way to check the encapsulation and isolation of the coil by the cover material during an electrophysiology experiment.

### 2.4. Activating function for estimating the site of activation by the figure-eight micro-coil

The component of the electric field gradient along the axon, or the activating function (Rattay, 1989), represents the driving force for activation of the axon. It defines the location and speed of depolarization or hyperpolarization by the extracellular stimulation (Rattay, 1986; Lee and Fried, 2017). In μMS technology, the activating function has been a powerful tool in estimating the site of axonal activation (Lee et al., 2016). When deriving the activating function for the figure-eight micro-coil, we considered the biphasic waveform of the induced electric field, since both the onset and offset phases of the stimulation pulse could play essential roles in axonal activation.

For the onset of a positive pulse, the activating function (gradient of electric field) in the direction of the axon was,


(10-1) AF=∂⁡Ex∂⁡x=Vμ0NRc2Ll{y(x+Rc)[(x+Rc)2+y2]2-y(x-Rc)[(x-Rc)2+y2]2}e-tRL

For the offset of the pulse, the activating function (gradient of electric field) in the direction of the axon was,


(10-2) AF=∂⁡Ex∂⁡x=-Vμ0NRc2Ll{y(x+Rc)[(x+Rc)2+y2]2-y(x-Rc)[(x-Rc)2+y2]2}e-tRL

Equations were derived and result figures were illustrated with Mathematica (version 12.3, Wolfram).

### 2.5. Combining the figure-eight micro-coil model with the axonal model in NEURON simulation

For NEURON simulation, we need to provide NEURON with the electric potential distribution along the modeled axon. This was calculated by integrating the scalar component of the electric field (Eqs. 6-1, 9-1) along the path of the axon. For the onset of a positive stimulation pulse, the electric potential distribution along the axon was,


(11-1) V(x)=∫Ex(x)dx=-Vμ0NRc22Ll

$$\;\text{[atan}\left(\frac{x+Rc}{y}\right)-\text{atan}\left(\frac{x-Rc}{y}\right)]e{}^{-\frac{tR}{L}}$$

For the offset of the pulse, the electric potential distribution along the axon was,


(11-2) V(x)=∫Ex(x)dx=Vμ0NRc22Ll

$$\;\text{[atan}\left(\frac{x+Rc}{y}\right)\text{atan}\left(\frac{x-Rc}{y}\right)]e{}^{-\frac{tR}{L}}$$

During NEURON simulation, the figure-eight micro-coil was positioned at the middle point of the axon (Left coil center: *x* = 10,000 μm; Right coil center: *x* = 10,500 μm), with the center of the coil 300 μm away from the axon (Figure 1B). Resting membrane potential was set to be −65 mV at the beginning of the simulation. The model was ran at 20 degrees Celsius. 250 ms after the initiation of the simulation, a single pulse (2.5 ms in duration) was sent to the coil for axonal activation. The electric voltages induced by the figure-eight micro-coil were used to create the extracellular stimuli. The waveform was defined as biphasic short pulses (1 ms in duration) with alternating directions, as measured experimentally (Figure 2). We defined a vector to store the waveform for each time step during stimulation. In each step of the simulation, the value of the extracellular potential was updated using “e_extracellular” at each compartment using the vector class’ “play” method (Joucla et al., 2014). Supplementary Figure 2 compared the difference between the extracellular potential for the figure-eight micro-coil and the circular micro-coil (Ye, 2022). Threshold of axonal activation was defined as the least coil voltage (V) that could initiate an action potential in the axon.

The parameters of the inductor provided by the manufacturer were used in this model, including the length of the coil (*l* = 0.5 mm), inductance of the coil (*L* = 100 nH), and resistance of the coil (*R* = 2 Ω). Each circular coil has a radius of *R_c_* = 0.25 mm. μ_0_ = 4π × 10^–7^ H/m.

## 3. Results

### 3.1. Temporal and spatial characterization of the electric field generated by a figure-eight micro-coil

Under time-varying magnetic stimulation with a magnetic coil, neural tissue is activated by the magnetically induced electric field *via* electromagnetic induction (Walsh and Pascual-Leone, 2003; Ye and Steiger, 2015). The temporal and spatial properties of the induced electric field are directly responsible for the activation of neural tissue with the micro-coil technology (Bonmassar et al., 2012; Lee and Fried, 2014). In most experimental settings, a single pulse or trains of electric pulses were delivered to the coil for neural activation. Due to electromagnetic induction theory, the electric field will be induced at the onset and offset of the stimulus pulse, when coil current is dramatically changed in a short period of time. Therefore, the electric field induced by the micro-coils are normally biphasic in shape, as has been measured by us (Ye and Barrett, 2021; Ye, 2022) and others (Lee and Fried, 2017; Golestanirad et al., 2018; Minusa et al., 2018).

For the figure-eight micro-coil, we have derived the analytical expression of the induced electric field (Eqs. 9-1, 9-2). Similar to a circular micro-coil, if a voltage pulse were to be delivered to the figure-eight micro-coil, the induced electric field is also a biphasic signal, triggered at the onset and offset of the single stimulus pulse. To validate this model prediction, we designed and implemented a figure-eight micro-coil using two commercial inductors that have been previously used for micromagnetic stimulation of various neural tissues (Bonmassar et al., 2012; Park et al., 2013; Ye et al., 2020; Ye and Barrett, 2021). The internal structure of the coil (Supplementary Figure 1) was revealed by chemically dissolving the cover of the inductor (Ye et al., 2020). We measured the induced electric field around the micro-coil using an electrophysiology setup. When a pulse signal of varying duration was delivered to the micro-coil, the shape of the induced electric field was biphasic in shape (Figure 2).

**FIGURE 3:**
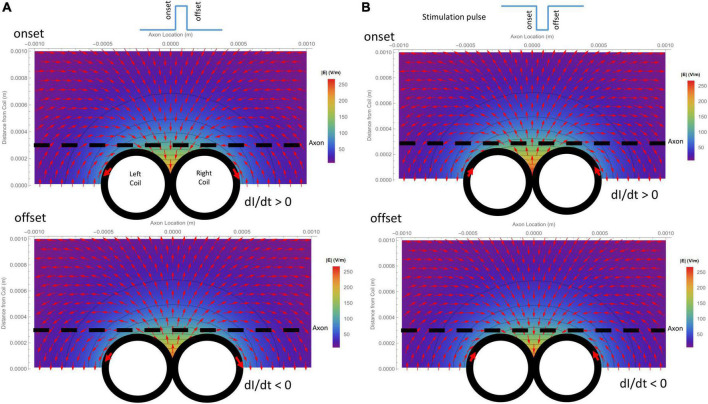
Spatial distribution of the induced electric field generated by a figure-eight micro-coil at the onset and offset of the stimulus pulse, respectively. Vector plot and contour plot were illustrated. The dashed black line represented the axon, located 300 μm away from the center of the coil. **(A)** Electric field generated by a positive voltage pulse in the coil. Coil current (I) was counterclockwise in the L-coil, and clockwise in the R-coil. The coil current was dramatically increased at the onset and decreased at the offset of the positive pulse. **(B)** Electric field generated by a negative voltage pulse in the coil. Coil current (I) was clockwise in the L-coil, and counterclockwise in the R-coil. The coil current dramatically increased at the onset and decreased at the offset of the negative pulse.

Due to its submillimeter scale, it was difficult to measure the spatial distribution of the induced electric field generated a micro-coil with conventional electrodes. We, therefore, computed and visualized the electric field generated by the figure-eight micro-coil (Figure 3). Figure 3A illustrates the electric field generated by the onset and offset of a positive stimulus pulse, respectively. Figure 3B illustrates the field generated by the onset and offset of a negative stimulus pulse, respectively. The coil currents in each of the two circular micro-coils have opposite directions. The intensity of the electric field was the summation of the two individual, circular micro-coils. This generated a large local field around the middle line of the figure-eight micro-coil. This field decayed quickly with increased distance from the coil.

### 3.2. Activating function predicts the location of membrane polarization and its dependency on coil parameters

**FIGURE 4:**
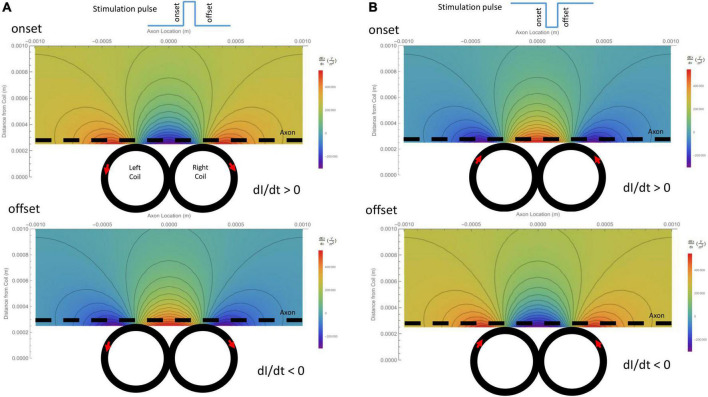
Gradient of the induced electric field (activating function) generated by the figure-eight micro-coil in the axon direction (*dEx/dx*), and its dependency on the coil parameters. Vector plot and contour plot were illustrated. The dashed black line represented the axon, located 300 μm away from the center from the coil. **(A)** Activating function generated by a positive voltage pulse in the coil. Coil current (I) was counterclockwise in the L-coil, and clockwise in the R-coil. The coil current was dramatically increased at the onset and decreased at the offset of the positive pulse. **(B)** Activating function generated by a negative voltage pulse in the coil. Coil current (I) was clockwise in the L-coil, and counterclockwise in the R-coil. The coil current was dramatically increased at the onset and decreased at the offset of the negative pulse.

The activating function is defined as the gradients of the electric field along the axon (Rattay, 1986). It predicts the location and speed of depolarization or hyperpolarization by the extracellular stimulation (Lee et al., 2016, 2019; Lee and Fried, 2017). The activating function generated by the figure-eight micro-coil was derived in (Eqs. 10-1, 10-2). Figure 4A illustrates the activating function (*dEx/dx*) generated by the onset and offset of a positive pulse, respectively. Figure 4B illustrates the activating function (*dEx/dx*) generated by the onset and offset of a negative pulse, respectively. The gradient of the induced electric field is at its maximum around the midline between the two coils and at the flank of the individual coils with opposite sign.

Previously, we have calculated the activating function of a circular micro-coil (Ye, 2022). This activating function allowed us to analyze the key factors that could cause shifting of activating location under circular micro-coil stimulation (Ye, 2022). Here, we took a similar approach to understand the location of axonal activation by a figure-eight micro-coil.

**FIGURE 5:**
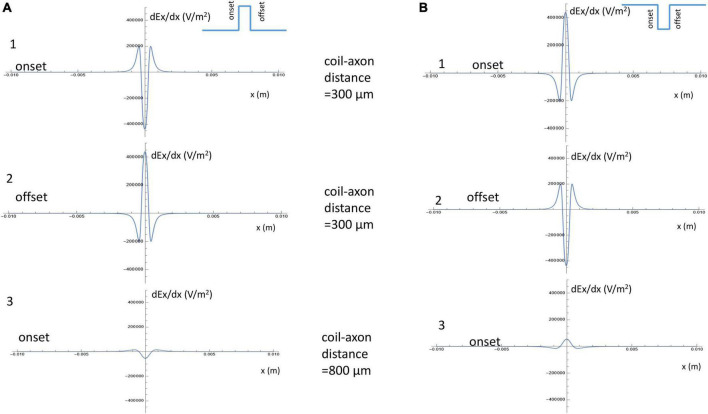
Activating function along the axon and its dependency on the coil parameters. **(A)** Activating function along the axon when a positive pulse was delivered into the figure-eight micro-coil. (1) Activating function at the onset of the positive pulse, when the coil-axon distance was 300 μm. (2) Activating function at the offset of the positive pulse, when the coil-axon distance was 300 μm. (3) Activating function at the onset of the positive pulse, when the coil-axon distance was 800 μm. **(B)** Activating function along the axon when a negative pulse was delivered into the figure-eight micro-coil. (1) Activating function at the onset of the negative pulse, when the coil-axon distance was 300 μm. (2) Activating function at the offset of the negative pulse, when the coil-axon distance was 300 μm. (3) Activating function at the onset of the negative pulse, when the coil-axon distance was 800 μm.

For a positive pulse, at its onset, the figure-eight micro-coil generated an activating function that contained a large “hyperpolarization peak” (virtual cathode) between the two coils (*x* = 0), and two smaller “depolarization peaks” (virtual anode, Figure 5A1). At the offset, the direction of the induced electric field was reversed, leading to the switch of the depolarization/hyperpolarization sites on the axon (Figure 5A2). For a negative pulse, at its onset, the figure-eight micro-coil generated an activating function that contained a large “depolarization peak” (virtual anode) between the two coils (*x* = 0), and two smaller “hyperpolarization peaks” (virtual cathode, Figure 5B1). At the offset, the direction of the induced electric field was reversed, leading to the switch of the depolarization/hyperpolarization sites on the axon (Figure 5B2). To estimate the impact of changes in the coil-axon distance on the site of activation, we increased the coil-axon distance from 300 to 800 μm. This led to a much smaller measurement of the activating function (Figures 5A3, 5B3). However, the large peaks in the activating functions did not change their locations.

These analyses allow us to make the following four predictions about axonal activation with a figure-eight micro-coil: (1) Since the sharp, larger depolarization peaks on the axon under figure-eight micro-coil stimulation are generated by the summation effects of two individual, circular micro-coils, the figure-eight micro-coil will need less coil voltage to initiate action potentials than a circular micro-coil. (2) Reversal of the stimulation pulse in the figure-eight micro-coil will reverse the depolarization/hyperpolarization sites, which may produce different mechanisms of axonal activation. (3) Increasing the distance between the figure-eight micro-coil and the axon will not change the activation site. (4) Increasing stimulus intensity will not cause shifting of the activation site. In the following sections, we will use a multi-compartment model of an unmyelinated axon to test these predictions in NEURON.

### 3.3. NEURON modeling of axonal activation with a figure-eight micro-coil demonstrates two types (A and B) of axonal activation, caused by the reversal of the coil current

A multi-compartment NEURON model of an unmyelinated axon (Figure 1B) was built to test the accuracy of these predictions. The figure-eight micro-coil is located in the middle segment of the axon. The distance between the center of the coil (O_1_ or O_2_) and the axon is *y* = 300 μm. In NEURON simulation, a single pulse (2.5 ms) was sent to the coil model. The model computed the voltage distribution along the axon (Eqs. 11-1, 11-2) and applied this information to the NEURON simulation environment (Supplementary Figure 2). This externally applied voltage triggered an action potential.

Previously, we have investigated the activation of the unmyelinated axon by a circular micro-coil (Figure 1A). We found two distinct ways in which the circular micro-coil could trigger an action potential in the axon (Ye, 2022). In Type I activation, the onset phase preconditioned the membrane for activation, and the offset phase of the stimulus pulse could trigger an action potential [Figure 6 in Ye (2022)]. This type of activation needs less stimulus intensity, with an activating function of 250,000 V/m^2^. For Type II activation, the onset phase of the stimulus pulse could directly trigger an action potential [Figure 8 in Ye (2022)]. This type of axonal activation requires a relatively larger stimulus intensity.

For the figure-eight micro-coil, depending on the direction of the coil current, we also observed two different types of axonal activation (Type A and Type B activations).

#### 3.3.1. Type A activation by a figure-eight micro-coil

**FIGURE 6:**
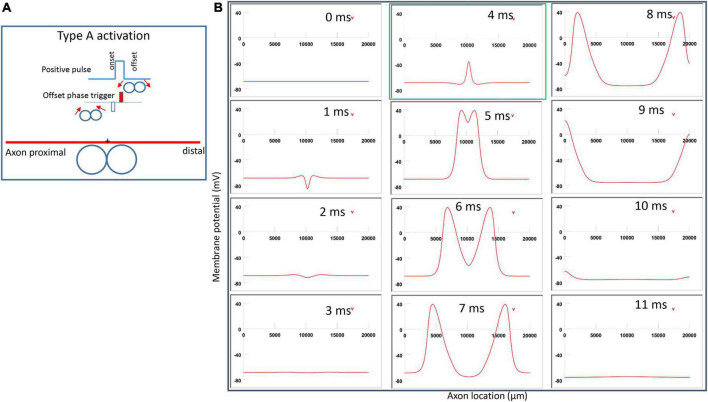
Activation of an unmyelinated axon by the figure-eight micro-coil with a positive pulse (Type A activation). The action potential was triggered by the offset phase of a positive stimulation pulse. **(A)** Coil current, direction of the induced electric field, and location of activation (+). The coil center was located 300 μm away from the axon. **(B)** A sequence of movie clips demonstrated the initiation and propagation of an action potential. Onset of the pulse generated a local hyperpolarization of the membrane, and the offset phase of the pulse generated a local depolarization, which triggered an action potential. The action potential was initiated at the midline between the two coils, and propagated in both directions. The green box, which shows the location of activation, will be used for comparison in the following Figures 7, 9, 11.

Figure 6A demonstrated a positive pulse applied to the figure-eight micro-coil, the induced electric field, and the location of axonal activation. Figure 6B simulated the initiation and traveling of an action potential with a sequence of frames (1 ms intervals). The onset of the pulse generated two depolarization peaks and one larger hyperpolarization peak (*t* = 1 ms in Figure 6B), as predicted with activating function analysis (Figure 5A1). The small depolarization peaks failed to trigger an action potential. Instead, an action potential was triggered during the subsequent offset phase of the coil current (*t* = 5 ms, Figure 6B), by depolarizing the membrane patch where the hyperpolarization peak presented during the onset phase. We refer to this activation pattern as Type A activation. In Type A activation, axonal activation happens at the membrane patch between the two circular micro-coils, due to membrane hyperpolarization followed by depolarization. The onset of the positive pulse conditions the membrane, and the axonal activation is eventually triggered by the offset phase. The action potential traveled bi-directionally to each end of the axon. The threshold for Type A activation by the figure-eight micro-coil is 83.5% of that required for Type I activation by the circular micro-coil (confirms prediction 1).

**FIGURE 7:**
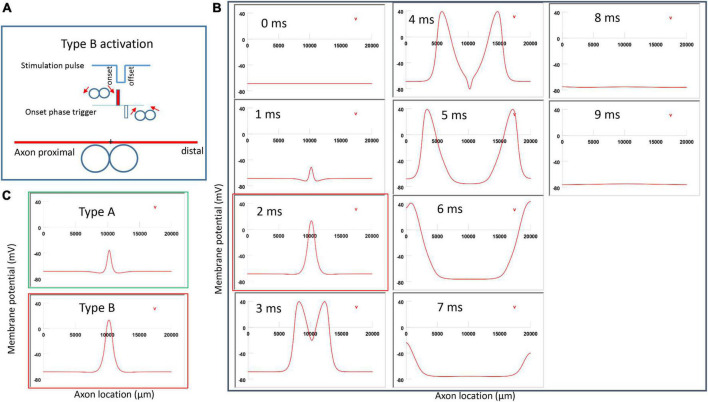
Activation of an unmyelinated axon by the figure-eight micro-coil (Type B activation) with a negative pulse. The action potential was triggered by the onset phase of a negative stimulation pulse. **(A)** Coil current, direction of the induced electric field, and location of activation (+). The coil center was located 300 μm away from the axon. **(B)** A sequence of movie clips demonstrated the initiation and propagation of an action potential. The action potential was initiated by the onset phase of the coil current, at the midline between two circular micro-coils, and propagated in both directions. The red box, which shows the location of activation, will be used for comparison in the following Figures 10, 12. **(C)** Comparison of locations of activation in Type A activation (Figure 6, green box) and Type B activation (red box) revealed no shifting of the activation sites.

**FIGURE 8:**
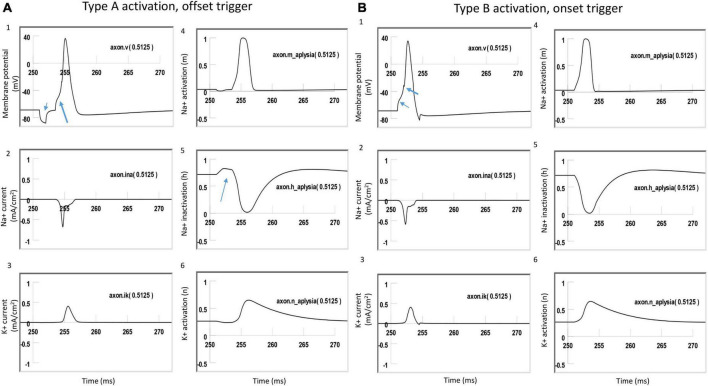
Comparison of ion channel dynamics for action potential initiation by Type A and Type B activations with a figure-eight micro-coil in an unmyelinated axon. Membrane potential (1), Na^+^ current (2), K^+^ current (3), Na^+^ channel activation m (4), Na^+^ channel inactivation h (5), and K^+^ channel activation n (6) were plotted at the locations where action potentials were initiated [axon (0.5125)]. **(A)** For Type A activation, the induced electric field during the onset phase hyperpolarized the cell membrane (thin arrow in A1) and de-inactivated the sodium channels (arrow in A5). Action potential was then initiated in the offset phase, which depolarized the membrane (thick arrow in A1). **(B)** For Type B activation, the induced electric field during the onset phase initiated the action potential simply by depolarizing the axonal membrane (thin arrow in B1) and activating the sodium channels. The offset phase did slightly hyperpolarize the membrane (thick arrow in B1), but it did not significantly affect the initiation and propagation of the action potential.

**FIGURE 9:**
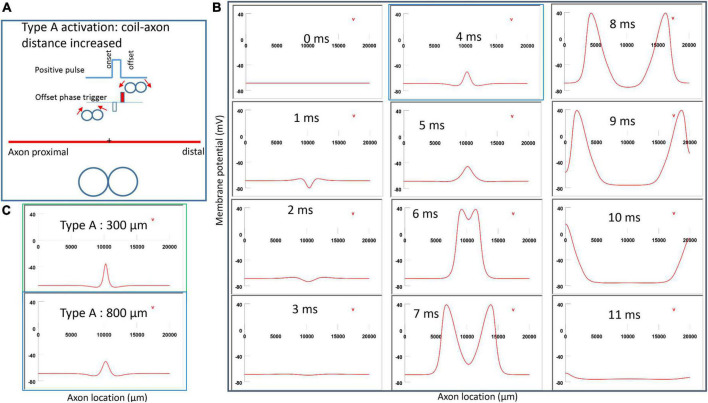
Impact of axon-coil distance on the location of Type A activation with a figure-eight micro-coil. **(A)** Coil current, direction of the induced electric field, and location of activation (+). The coil-axon distance was increased from 300 μm (in Figure 6) to 800 μm. Stimulation intensity was adjusted to trigger the action potential. **(B)** A sequence of movie clips demonstrated the initiation and propagation of an action potential. The blue box showed the location of activation. **(C)** Comparison of locations of Type A activation when the axon-coil distance was 300 μm (Figure 6, green box) and 800 μm (blue box). Increase in coil-axon distance did not significantly change the location of activation in Type A activation.

**FIGURE 10:**
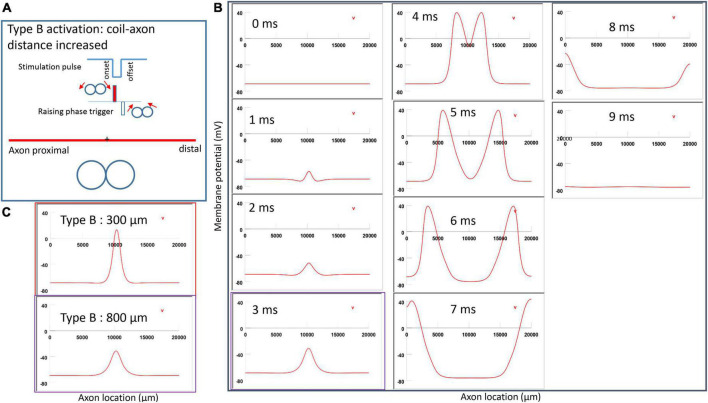
Impact of axon-coil distance on the location of activation in Type B activation with a figure-eight micro-coil. **(A)** Coil current, direction of the induced electric field, and location of activation (+). The coil-axon distance was increased from 300 μm (Figure 7) to 800 μm. Stimulation intensity was adjusted to trigger action potentials. **(B)** A sequence of movie clips demonstrated the initiation and propagation of an action potential. The purple box showed the location of activation. **(C)** Comparison of locations of Type B activation when the axon-coil distance was 300 μm (Figure 7, red box) and the 800 μm (purple box). Increase in coil-axon distance did not significantly change the location of activation in Type B activation.

**FIGURE 11:**
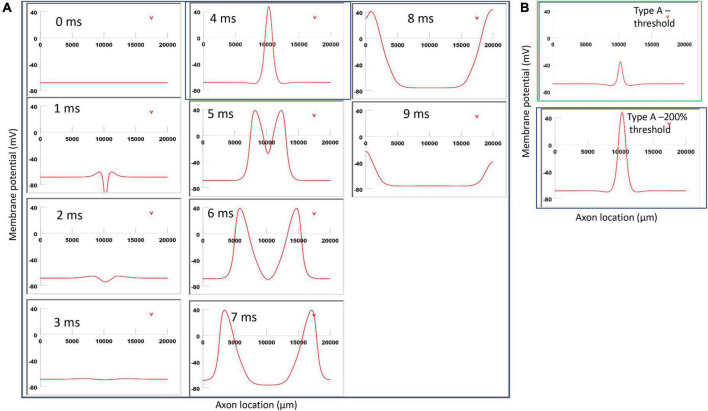
Impact of stimulation intensity on the location of Type A activation. **(A)** A strong Type A activation with 200% threshold intensity was applied to the axon. A sequence of movie clips demonstrated the initiation and propagation of the action potential. The black box showed the location of activation. **(B)** Comparison of the locations of activation with threshold stimulation (Figure 6, green box) and the black box when the stimulation intensity was doubled. Increasing stimulation intensity caused a quicker axonal activation, but it did not change the location of activation in Type A activation.

**FIGURE 12:**
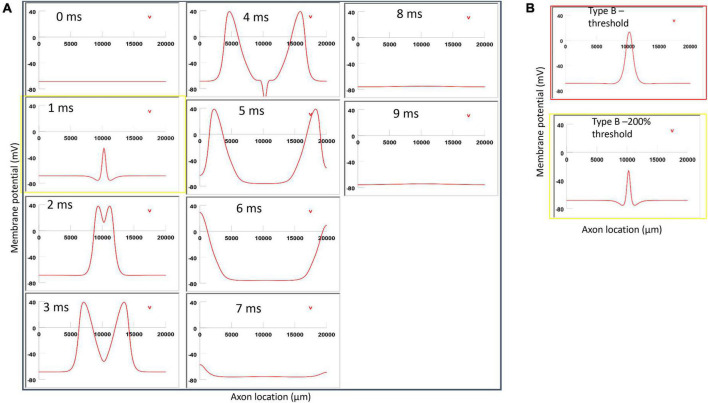
Impact of stimulation intensity on the location of Type B activation. **(A)** A strong Type B activation with 200% threshold intensity was applied to the axon. A sequence of movie clips demonstrated the initiation and propagation of the action potential. The yellow box showed the location of activation. **(B)** Comparison of the locations of activation with threshold stimulation (Figure 7, red box) and the yellow box when the stimulation intensity was doubled. Increasing stimulation intensity caused a faster activation, but it did not change location of activation in Type B activation.

#### 3.3.2. Type B activation by a figure-eight micro-coil

Figure 7A demonstrated a negative pulse applied to the figure-eight micro-coil, the induced electric field, and the location of axonal activation. Figure 7B simulated the initiation and traveling of an action potential with a sequence of frames (1 ms intervals). The onset of the pulse generated two small hyperpolarization peaks and one larger depolarization peak (*t* = 1 ms in Figure 7B), as predicated with activating function analysis (Figure 5B1). The large depolarization peak triggered an action potential during the onset phase of the coil current (*t* = 3 ms, Figure 7B). The offset phase of the pulse generated a local hyperpolarization (*t* = 4 ms, Figure 7B), but it did not affect the action potential. In Type B activation, axonal activation occurred at the membrane patch between the two circular micro-coils due to a strong depolarization. The action potential traveled bi-directionally to each end of the axon. We refer to this activation pattern as Type B activation, in which the onset phase of a negative pulse directly activates the axon by membrane depolarization. Since Type B activation is triggered by the onset of the stimulation pulse, axonal activation was faster in Type B activation than in Type A activation. The threshold for Type B activation is slightly (3.7%) higher than Type A activation. The threshold for Type B activation by the figure-eight micro-coil is 46.8% of that required for Type II activation by the circular micro-coil (confirms prediction 1).

In conclusion, reversal of the stimulation pulse in the figure-eight micro-coil will reverse the depolarization/hyperpolarization sites, which produces two distinct types of axonal activation (confirms prediction 2). However, reversal of pulse polarity did not cause the activation site to shift. The axon was activated at the same location in both Type A and Type B activations (Figure 7C), which ensures that the consistency of stimulation is not affected by accidental reversal of coil current.

### 3.4. Distinct ion channel mechanisms underlying type A vs. type B axonal activation by a figure-eight micro-coil

To further investigate the ionic mechanism underlying action potential initiation in Type A and Type B activations with the figure-eight micro-coil, we studied the membrane dynamics at the locations where action potentials were initiated. We compared the inward sodium current (INa+), outward potassium current (IK+), sodium channel activation (m) and inactivation (h) variables, and potassium channel activation (n) variables. The center of the two coils were at (10,000 μm, −300 μm) and (10,500, −300 μm), respectively. We found that the largest depolarization happened at *x* = 10, 250 μm, which was the midpoint between the two micro-coils. In NEURON, this point is Axon (0.5125). We, therefore, plotted the channel dynamics at this point in both Type A and B activations. Type A and Type B activations with the figure-eight micro-coil demonstrated different ionic mechanisms for triggering action potentials.

Figure 8A demonstrated Type A activation, in which the membrane patch experienced a brief hyperpolarization during the onset phase of the positive stimulation pulse, followed by a depolarization induced by the offset phase of the stimulation pulse, which eventually triggered an action potential (Figure 8A1). The initial hyperpolarization did not cause significant changes in the state variable *m* (around 0, Figure 8A4) and *n* (around 0.3, Figure 8A6) values. However, the hyperpolarization removed sodium channel inactivation and caused a clear increase in the *h* value (from 0.7 to 0.8, Figure 8A5). This de-inactivation of the sodium channels led to an increase in the sodium conductance, defined as m3h (Hodgkin and Huxley, 1952). The large *h* value was maintained after the termination of the onset phase until the offset phase, which caused a membrane depolarization, leading to a large inward sodium current (Figure 8A2) and action potential initiation (Figure 8A1). Due to the hyperpolarization during the onset phase, a larger amount of sodium channels were preconditioned for activation during the offset phase, which is why less stimulation intensity was needed in Type A activation than in Type B activation.

Figure 8B demonstrated Type B activation, in which the membrane patch experienced a large depolarization during the onset phase of the stimulation pulse (Figure 8B1). The depolarization mainly activated the sodium channels by increasing the *m* value (from 0 to 0.3, Figure 8B4). This increase in sodium channel activation led to the initiation of an action potential. In comparison with Type A activation (Figure 8A5), this depolarization did not alter the *h* value (Figure 8B5) to de-inactivate more sodium channels. The following offset phase introduced a brief membrane hyperpolarization; however, it did not affect the action potential.

In summary, under Type A activation, the local membrane was hyperpolarized during the onset phase of the coil current, which de-inactivated the sodium channels. This same membrane patch was then activated by the offset phase of the coil current, and an action potential was initiated. In contrast, during Type B activation (which requires greater coil current than Type A activation), the action potential was generated at the same location, where the membrane was directly depolarized by the onset phase of the coil current.

### 3.5. Activation location does not change with varying coil-axon distance

Previously, when modeling the circular micro-coil, we found that changing the coil-axon distance could cause shifting of the activation site [Figure 7 in Ye (2022)]. Activating function analysis for the figure-eight micro-coil, in contrast, suggests that the site of membrane depolarization/hyperpolarization is not affected by the increase in coil-axon distance in Type A (Figure 5A3) or Type B (Figure 5B3) activations.

In NEURON simulation, we increased the coil-axon distance from 300 to 800 μm and pinpointed the site where the action potential was initiated. For both Type A (Figures 9A, B) and Type B (Figures 10A, B) activations, a greater stimulation intensity was required to trigger an action potential. However, increasing the coil-axon distance caused no shifting of the activation site in Type A activation (Figure 9C). Similarly, increasing the coil-axon distance caused no shifting of the activation site in Type B activation (Figure 10C) (confirms prediction 3).

### 3.6. Location of activation does not change with varying stimulation intensities

Previously, when modeling the single, circular micro-coil, we found that the activation site was dependent on the intensity of coil stimulation [Figure 8 in Ye (2022)]. Activating function analysis for the figure-eight micro-coil, in contrast, suggests that the site of membrane depolarization/hyperpolarization was not affected by the intensity of the stimulus (Eqs. 10-1, 10-2). We hypothesize that increasing the stimulus intensity will not cause any shifting in the activation sites for both Type A and Type B activation.

In Figure 11, for Type A activation, we doubled the stimulation intensity in the figure-eight micro-coil. Onset of the pulse generated a local hyperpolarization, followed by a depolarization and the initiation of the action potential, which traveled in both directions along the axon (Figure 11A). In comparison to Figure 6, local depolarization and hyperpolarization were more prominent when the stimulus intensity doubled, leading to a quick initiation of the action potential. However, the location of activation was not shifted by the intense stimulation in Figure 11B (confirms prediction 4).

For Type B activation, we doubled the stimulation intensity in the figure-eight micro-coil. Onset of the pulse generated a large local depolarization and the initiation of the action potential, which traveled in both directions along the axon (Figure 12A). In comparison to Figure 7, this local depolarization was more prominent when the stimulus intensity was doubled, leading to a quicker initiation of the action potential. However, the location of activation was not shifted by the intense stimulation in Figure 12B (confirms prediction 4).

In summary, NEURON simulation confirms the four predictions made in the activating function analysis. With less coil current, the figure-eight micro-coil provides a more focal activation than the circular micro-coil. This activation site does not shift by the reversal of the coil current, although it will result in different mechanisms of axonal activation. The location of axonal activation by the figure-eight micro-coil is not affected by the intensity of coil current or the translational movement of the coil away from the axon. These results suggest that the figure-eight micro-coil could provide significantly improved consistency over the circular micro-coil in axonal activation.

## 4. Discussion

One of the major goals of magnetic stimulation with a micro-coil is to provide consistent stimulation of individual neurons. Previously, we have investigated the consistency of neural simulation with a circular micro- coil, using combined biophysics modeling and NEURON simulation (Ye, 2022). This paper further examines the consistency of micro-coil stimulation with a figure-eight micro-coil, and compares it with the circular micro-coil. The figure-eight micro-coil provides significantly improved consistency in neural stimulation. Table 1 compares axonal activation by a figure-eight micro-coil and a circular micro-coil [detailed in Ye (2022)], respectively.

**TABLE 1:** Comparison between the circular micro-coil and figure-eight micro-coil in axon activation.

Coil type	Circular	Circular	Figure–eight	Figure–eight
**Type of activation**	Type I	Type II	Type A	Type B
**Stimulus signal**	Single pulse (positive or negative)	Single pulse (positive or negative)	Single pulse (positive)	Single pulse (negative)
**Waveform of induced electric field**	Biphasic (onset and offset phases)	Biphasic (onset and offset phases)	Biphasic (onset and offset phases)	Biphasic (onset and offset phases)
**Action potential triggering time**	Offset phase	Onset phase	Offset phase	Onset phase
**Membrane dynamic responsible for action potential initiation**	Hyperpolarization–depolarization	Depolarization	Hyperpolarization–depolarization	Depolarization
**Ion channel dynamics underlying action potential initiation**	Na channel de-inactivation followed by activation	Na channel activation	Na channel de-inactivation followed by activation	Na channel activation
**Activation site:** **Coil current direction dependent**	Yes	Yes	No	No
**Activation site:** **Coil axon distance dependent**	Yes	Yes	No	No
**Activation site:** **Coil current intensity dependent**	Yes	Yes	No	No
**Threshold of activation** **a. Same coil comparison** **b. Different coil comparison**	250,000 V/m^2^ (Ye, 2022). − −	184.6% of Type I −	− 83.5% of Type I	103.7% of Type A 46.8% of Type II

For detailed analysis of the circular micro-coil, see reference Ye (2022).

First, a single stimulus pulse delivered to either of these coils generates a biphasic electric field, due to the quick coil current changes at the onset and offset phases of the stimulus pulse. The biphasic waveform is a favored waveform when an electrode is used to deliver currents for neural blockage in peripheral nerves (Cattell and Gerard, 1935; Tanner, 1962). When compared to monophasic stimulation, biphasic stimulation causes less tissue damage due to the neutralization properties of electrochemical reactions (Tai et al., 2005). The intrinsic biphasic nature of the induced electric field suggests that micro-coil stimulation can lead to a null charge integrated over time, and provide biocompatible stimulation (Jeong et al., 2022).

Second, both the figure-eight micro-coil and circular micro-coil could activate the axon *via* two distinct mechanisms. These activation patterns share many commonalities. In Type I (circular micro-coil) and Type A (figure-eight micro-coil) activations, the membrane is hyperpolarized at the onset phase, and the sodium channels are sufficiently de-inactivated. Action potential initiation occurs during the offset phase, which depolarizes the membrane. In Type II (circular) and Type B (figure-eight) activations, the membrane is depolarized at the onset phase, with sufficiently inactivates the sodium channels and elicits the action potential. As the consequence of ion channel preconditioning, the threshold for Type I (circular) and Type A (figure-eight) activations are significantly lower than those in Type II (circular) and Type B (figure-eight) activations, respectively.

Third, the figure-eight micro-coil provides a much more focal and consistent axonal activation mechanism than the circular micro-coil. In circular micro-coil stimulation, the activation site shifts with the reversal of coil current direction, changes in the coil-axon distance, and increases in the coil current intensity. In figure-eight micro-coil stimulation, however, changes in these experimental factors have a minimal impact on the site of activation.

Finally, stimulation with the figure-eight micro-coil, overall, requires less coil current than the circular micro-coil for axonal activation. Furthermore, activation due to the preconditioning of the sodium channel (Type I and Type A activations) normally require less coil current than activation without such preconditioning (Type II and Type B activations). These results could potentially provide a solution to the heating effects observed in some μMS practices (Ye and Barrett, 2021).

### 4.1. Implication to the development of μMS technology

Results from this work provide several insights to the novel design of micro-coils for efficient neural stimulation with improved consistency.

First, figure-eight micro-coil design could provide a simple design strategy to improve stimulating efficacy. In circular micro-coil stimulation, to increase the intensity of the induced electric field for stimulation, one could include more winding or encapsulate a core with high magnetic permeability. This could potentially increase the size of the coil and provide challenges in implantation and increase the risk of brain damage. This problem could be avoided, however, if a figure-eight micro-coil were to be used for neural stimulation. A recent work simulated the micro-coil stimulation of the radial nerve fibers in arms with a circular micro-coil and a figure-eight micro-coil. The authors found that the threshold current in the figure-eight micro-coil was significantly less than the circular micro-coil for neural activation (Colella et al., 2021). Our results are generally in agreeance with this report. The figure-eight coil could generate a large local field gradient without the need to significantly increase the winding and coil current.

Second, it is essential to have a detailed understanding of both spatial and temporal properties of the electric field generated by the figure-eight micro-coil. Our results demonstrate that these properties play significant roles in the pattern of membrane depolarization/hyperpolarization (Figures 6, 7), which define the site and speed of axonal activation. Various technologies are available to measure the electric field generated by the micro-coil. This includes electrophysiological recording electrodes to measure the field potential (Lee and Fried, 2017; Golestanirad et al., 2018), custom-made pickup coils to measure the electromotive force (Minusa et al., 2018), and nitrogen vacancy in diamond magnetometers to directly measure the magnetic flux density (Khalifa et al., 2021). The spatial resolution of these measurements could be limited by the size of the sensors. Alternatively, finite element modeling provides an easy estimation of the induced electric field with high resolution (Lee et al., 2019; Saha et al., 2022a).

Third, it is essential to design the stimulus waveforms and understand their significance in neural stimulation using μMS technology. Previously, using a circular micro-coil as a model, we have demonstrated the importance of the stimulus waveform in affecting the location of axonal activation (Ye, 2022). Here, using a figure-eight micro-coil, we demonstrated that, depending on the direction of coil current, axonal activation could be triggered by either the offset of the pulse (Type A, Figure 6) or onset of the pulse (Type B, Figure 7). Therefore, deliberate control of coil current could speed up or delay the onset time of action potentials.

Fourth, it is essential to understand the biophysics mechanisms of neural activation when applying a stimulus. For example, the stimulus threshold for Type B activation is lower than Type A activation. This is because the membrane patch in Type A activation experienced a priming period during the onset phase of the stimulus pulse, leading to the de-inactivation of the sodium channels (Figure 8A). Therefore, when choosing a specific stimulation intensity needed for axonal activation, one must consider the properties of the neurons, and the dynamic interaction between the neuron and the field (Ye and Steiger, 2015). On the other hand, deliberate control of the stimulus could lead to reduced coil current, and prevent unnecessary heating effects associated with micro-coil technology (Saha et al., 2022b).

Finally, it is essential to further develop multi-coil technology. Determining the optimal configuration for efficient stimulation has been a long quest in neuroscience, both in fundamental research and for therapeutic development. One method is to combine the source of stimuli (Zaaimi et al., 2013; Alekseichuk et al., 2019). For example, in electrode stimulation, experimental strategies aiming to improve the stimulating focality around the activation sites generally combines two (Rattay and Resatz, 2007), three (Sankarasubramanian et al., 2011), or multiple electrodes (Zaaimi et al., 2013). Such multipolar electrodes are usually associated with higher activation thresholds than the monopolar, single electrode configuration. Since tissue damage and electrode deterioration are directly related to the injected electric current *via* the multiple electrodes, electric current intensity must be constrained for safety reasons (Gunter et al., 2019). In this paper, when two circular micro-coils were combined to construct the figure-eight micro-coil, we could obtain a significant increase in the focality and field gradient with a decreased current in the coil. Future research in μMS technology must explore the possibility of combining multiple micro-coils for highly specific stimulation.

### 4.2. Implication to neural activation with highly consistent μMS

Recent μMS research focuses on achieving high resolution stimulation at the cellular level. There have been several preparations in which μMS has been successful in neural activation. The goal of these works is to constrain the stimulation site within certain geometrical dimensions of the neural tissue. In order to achieve consistent stimulation results, it is essential to know the dimensions of the target tissue and estimate the allowable range of shifting in the activation sites.

One of the early μMS successes was stimulating retinal ganglion cells (RGCs) (Bonmassar et al., 2012). These cells can vary in soma size and dendritic field size, stratification, shape, and compactness (Kim et al., 2021). For example, midget RGCs, which functionally serve to distinguish red and green, have a dendritic field size range of 10–100 μm and, overall, have smaller cell bodies and receptive fields. Meanwhile, parasol RGCs, which have the function of movement, have a dendritic field size range of 30–300 μm and have larger cell bodies and receptive fields. RGC axons can drastically vary in length from 0.5 μm to 50 mm (Yu et al., 2013), and are typically myelinated in the optic nerve and unmyelinated in the retinal nerve fiber layer (Yang et al., 2013). Therefore, the size of the soma, dendritic field, and axon of RGCs as well as the function and myelination of the cell necessitates highly specific stimulation, most typically in the micrometer range. This ultimately requires delicate control with high specificity and consistency, which could potentially be achieved by the figure-eight micro-coil or another combined coil strategy.

μMS was also used to stimulate presynaptic axons in the Schaffer collateral for synaptic transmission in the hippocampus (Saha et al., 2022a). The synaptic cleft is 20–30 nm at synapses between neurons in the central nervous system and about 50 nm at the neuromuscular junction. A shifting of stimulation site could potentially miss the presynaptic site, rather activating the postsynaptic neurons. A figure-eight micro-coil could possibly provide a more reliable stimulation by ensuring the presynaptic axon is the sole target for stimulation. Alternatively, if a circular micro-coil were to be used, to avoid side effects of shifting in the activation site, it should be positioned far from the CA1 area where synaptic transmission occurs.

μMS was also used to stimulate apical dendrites of the cortex’s pyramidal neurons (PNs) without activating horizontally oriented passing axons in mice (Lee et al., 2016). In these experiments, the length of a single dendrite is usually several hundred micrometers. This is a dimension that is unlikely to be missed by the μMS technology.

## 5. Conclusion and future work

This paper demonstrates that figure-eight μMS could generate consistent axonal activation with high spatial resolution. Because the site of activation was unaffected by the changes in the direction and intensity of the coil current, and was likewise unaffected by the increase in coil-axon distance, neural stimulation using the figure-eight micro-coil could have a higher tolerance for human errors in clinical applications. The coil needs less intensive coil current for axonal activation, which could be a tremendous advantage in avoiding unwanted thermal effects associated with the μMS technology. Although simulation results coming from this work must be fully validated with future experiments, the obvious advantages provided by the figure-eight micro-coil advocate for further efforts in the design, manufacturing, and development of the figure-eight coil in μMS technology.

Previous studies have suggested that axonal bending (Maccabee et al., 1993) and the presence of dendritic/axonal terminals (Radman et al., 2009; Rahman et al., 2013) could be the target of electric field activation, which are not integrated in the current model. It would be interesting and clinically relevant to see if the figure-eight coil could provide focal and consistent neural activation when these morphological details are represented in the model. Finite element modeling would be a fitting tool to represent such complicated neural morphology. The excellent agreement between activating function analysis and the site of neural activation (provided by NEURON modeling) allows us to speculate that, if such complicated neural geometry should be considered, activating function analysis could still serve as a powerful tool to predict the site of neural activation.

Increased resolution of stimulation with the figure-eight micro-coil could have a significant impact on basic research. For example, microelectric stimulation on a single neuron has been used to alter the activity of large networks and animal behavior (Houweling and Brecht, 2008). Optogenetically driving a fraction of neurons in the mouse primary somatosensory cortex could control behavior in freely moving mice (Huber et al., 2008). These studies establish causal links between the activity of single neurons and perceptual and cognitive functions, as well as animal behavior. It is expected that future μMS technology could achieve single cell resolution, therefore providing a more biocompatible tool to selectively activate or inhibit neurons, and could provide causal evidence of the functional roles of specific neurons. Future work will test the specificity and consistency of single neuron stimulation by the figure-eight micro-coil configuration using *in vitro* or *in vivo* animal models.

## Data availability statement

The original contributions presented in this study are included in the article/Supplementary material, further inquiries can be directed to the corresponding author.

## Author contributions

HY and VH: biophysics modeling. HY: NEURON simulation. All authors manuscript drafting and read and approved the final manuscript.

## Supplementary material

Supplementary Figure 1 Figure-eight micro-coil used in neural stimulation experiments. The coil was assembled from two commercially available inductors. **(A)** Coil internal structure revealed by the chemical removal of the encapsulation. **(B)** Assembly of the figure-eight coil with the two inductors stacked on top of each other, with opposite wire winding.

Supplementary Figure 2 Extracellular potential along the modeled axon in NEURON simulation. The potential was generated by a positive pulse delivered through a circular micro-coil **(A)** versus a figure-eight micro-coil **(B)**.

## References

[B1] Ahmed S. I. Javed G. Mubeen B. Bareeqa S. B. Rasheed H. Rehman A. (2018). Robotics in neurosurgery: A literature review. *J. Pak Med. Assoc.* 68 258–263. 29479103

[B2] Alekseichuk I. Falchier A. Y. Linn G. Xu T. Milham M. P. Schroeder C. E. (2019). Electric field dynamics in the brain during multi-electrode transcranial electric stimulation. *Nat. Commun.* 10:2573. 10.1038/s41467-019-10581-7 31189931 PMC6561925

[B3] Barker A. T. Jalinous R. Freeston I. L. (1985). Non-invasive magnetic stimulation of human motor cortex. *Lancet* 1 1106–1107. 10.1016/s0140-6736(85)92413-4 2860322

[B4] Bohning D. E. Shastri A. McConnell K. A. Nahas Z. Lorberbaum J. P. Roberts D. R. (1999). A combined TMS/fMRI study of intensity-dependent TMS over motor cortex. *Biol. Psychiatry* 45 385–394. 10.1016/s0006-3223(98)00368-0 10071706

[B5] Bonmassar G. Lee S. W. Freeman D. K. Polasek M. Fried S. I. Gale J. T. (2012). Microscopic magnetic stimulation of neural tissue. *Nat. Commun.* 3:921. 10.1038/ncomms1914 22735449 PMC3621430

[B6] Cattell M. Gerard R. W. (1935). The “inhibitory” effect of high-frequency stimulation and the excitation state of nerve. *J. Physiol.* 83 407–415. 10.1113/jphysiol.1935.sp003238 16994640 PMC1394415

[B7] Cogan S. F. (2008). Neural stimulation and recording electrodes. *Annu. Rev. Biomed. Eng.* 10 275–309. 10.1146/annurev.bioeng.10.061807.160518 18429704

[B8] Colella M. Liberti M. Apollonio F. Bonmassar G. (2021). “A miniaturized ultra-focal magnetic stimulator and its preliminary application to the peripheral nervous system,” in *Brain and human body modeling 2020: Computational human models presented at EMBC 2019 and the BRAIN initiative(R) 2019 meeting*, eds Makarov S. N. Noetscher G. M. Nummenmaa A. (Cham: Springer), 167–176. 10.1007/978-3-030-45623-8_9 32966015

[B9] Fernandes de Oliveira Santos B. de Araujo Paz D. Fernandes V. M. Dos Santos J. C. Chaddad-Neto F. E. A. Sousa A. C. S. (2021). Minimally invasive supratentorial neurosurgical approaches guided by Smartphone app and compass. *Sci. Rep.* 11:6778. 10.1038/s41598-021-85472-3 33762597 PMC7991647

[B10] Gluckman B. J. Neel E. J. Netoff T. I. Ditto W. L. Spano M. L. Schiff S. J. (1996). Electric field suppression of epileptiform activity in hippocampal slices. *J. Neurophysiol.* 76 4202–4205. 10.1152/jn.1996.76.6.4202 8985916

[B11] Golestanirad L. Gale J. T. Manzoor N. F. Park H. J. Glait L. Haer F. (2018). Solenoidal micromagnetic stimulation enables activation of axons with specific orientation. *Front. Physiol.* 9:724. 10.3389/fphys.2018.00724 30140230 PMC6094965

[B12] Gunter C. Delbeke J. Ortiz-Catalan M. (2019). Safety of long-term electrical peripheral nerve stimulation: review of the state of the art. *J. Neuroeng. Rehabil.* 16:13. 10.1186/s12984-018-0474-8 30658656 PMC6339286

[B13] Habibollahi Saatlou F. Rogasch N. C. McNair N. A. Biabani M. Pillen S. D. Marshall T. R. (2018). MAGIC: An open-source MATLAB toolbox for external control of transcranial magnetic stimulation devices. *Brain Stimul.* 11 1189–1191. 10.1016/j.brs.2018.05.015 29885859

[B14] Hines M. L. Carnevale N. T. (1997). The NEURON simulation environment. *Neural Comput.* 9 1179–1209. 10.1162/neco.1997.9.6.1179 9248061

[B15] Hodgkin A. L. Huxley A. F. (1952). A quantitative description of membrane current and its application to conduction and excitation in nerve. *J. Physiol.* 117 500–544. 10.1113/jphysiol.1952.sp004764 12991237 PMC1392413

[B16] Houweling A. R. Brecht M. (2008). Behavioural report of single neuron stimulation in somatosensory cortex. *Nature* 451 65–68. 10.1038/nature06447 18094684

[B17] Huber D. Petreanu L. Ghitani N. Ranade S. Hromadka T. Mainen Z. (2008). Sparse optical microstimulation in barrel cortex drives learned behaviour in freely moving mice. *Nature* 451 61–64. 10.1038/nature06445 18094685 PMC3425380

[B18] Jefferys J. G. (1981). Influence of electric fields on the excitability of granule cells in guinea-pig hippocampal slices. *J. Physiol.* 319 143–152. 10.1113/jphysiol.1981.sp013897 7320909 PMC1243827

[B19] Jeong H. Cho A. Ay I. Bonmassar G. (2022). Short-pulsed micro-magnetic stimulation of the vagus nerve. *Front. Physiol.* 13:938101. 10.3389/fphys.2022.938101 36277182 PMC9585240

[B20] Jeong H. Deng J. Bonmassar G. (2021). Planar figure-8 coils for ultra-focal and directional micromagnetic brain stimulation. *J. Vac. Sci. Technol. B Nanotechnol. Microelectron.* 39:063202. 10.1116/6.0001281 34692236 PMC8516478

[B21] Joucla S. Gliere A. Yvert B. (2014). Current approaches to model extracellular electrical neural microstimulation. *Front. Comput. Neurosci.* 8:13. 10.3389/fncom.2014.00013 24600381 PMC3928616

[B22] Khalifa A. Zaeimbashi M. Zhou T. X. Abrishami S. M. Sun N. Park S. (2021). The development of microfabricated solenoids with magnetic cores for micromagnetic neural stimulation. *Microsyst. Nanoeng.* 7:91. 10.1038/s41378-021-00320-8 34786205 PMC8589949

[B23] Kim U. S. Mahroo O. A. Mollon J. D. Yu-Wai-Man P. (2021). Retinal ganglion cells-diversity of cell types and clinical relevance. *Front. Neurol.* 12:661938. 10.3389/fneur.2021.661938 34093409 PMC8175861

[B24] Koivuniemi A. Wilks S. J. Woolley A. J. Otto K. J. (2011). Multimodal, longitudinal assessment of intracortical microstimulation. *Prog. Brain Res.* 194 131–144. 10.1016/B978-0-444-53815-4.00011-X 21867800 PMC8098704

[B25] Lee J. I. Seist R. McInturff S. Lee D. J. Brown M. C. Stankovic K. M. (2022). Magnetic stimulation allows focal activation of the mouse cochlea. *Elife* 11:e76682. 10.7554/eLife.76682 35608242 PMC9177144

[B26] Lee S. W. Fried S. I. (2014). The response of L5 pyramidal neurons of the PFC to magnetic stimulation from a micro-coil. *Conf. Proc. IEEE Eng. Med. Biol. Soc.* 2014 6125–6128. 10.1109/EMBC.2014.6945027 25571395 PMC4465444

[B27] Lee S. W. Fried S. I. (2017). Enhanced control of cortical pyramidal neurons with micromagnetic stimulation. *IEEE Trans. Neural Syst. Rehabil. Eng.* 25 1375–1386. 10.1109/TNSRE.2016.2631446 27893396 PMC5498237

[B28] Lee S. W. Fallegger F. Casse B. D. Fried S. I. (2016). Implantable microcoils for intracortical magnetic stimulation. *Sci. Adv.* 2:e1600889. 10.1126/sciadv.1600889 27957537 PMC5148213

[B29] Lee S. W. Thyagarajan K. Fried S. I. (2019). Micro-coil design influences the spatial extent of responses to intracortical magnetic stimulation. *IEEE Trans. Biomed. Eng.* 66 1680–1694. 10.1109/Tbme.2018.2877713 30369434 PMC6561646

[B30] Maccabee P. J. Amassian V. E. Eberle L. P. Cracco R. Q. (1993). Magnetic coil stimulation of straight and bent amphibian and mammalian peripheral nerve in vitro: locus of excitation. *J. Physiol.* 460 201–219. 10.1113/jphysiol.1993.sp019467 8487192 PMC1175209

[B31] Minusa S. Muramatsu S. Osanai H. Tateno T. (2019). A multichannel magnetic stimulation system using submillimeter-sized coils: system development and experimental application to rodent brain in vivo. *J. Neural. Eng.* 16:066014. 10.1088/1741-2552/ab3187 31642445

[B32] Minusa S. Osanai H. Tateno T. (2018). Micromagnetic stimulation of the mouse auditory cortex in vivo using an implantable solenoid system. *IEEE Trans. Biomed. Eng.* 65 1301–1310. 10.1109/TBME.2017.2748136 28880154

[B33] Osanai H. Minusa S. Tateno T. (2018). Micro-coil-induced Inhomogeneous electric field produces sound-driven-like neural responses in microcircuits of the mouse auditory cortex in vivo. *Neuroscience* 371 346–370. 10.1016/j.neuroscience.2017.12.008 29246784

[B34] Park H. J. Bonmassar G. Kaltenbach J. A. Machado A. G. Manzoor N. F. Gale J. T. (2013). Activation of the central nervous system induced by micro-magnetic stimulation. *Nat. Commun.* 4:2463. 10.1038/Ncomms3463 24030203 PMC3845906

[B35] Park Y. S. Kim J. Chang W. S. Chang J. W. (2011). Management of a DBS system in patients with traumatic brain injury: Case report. *Neuromodulation* 14 214–218; discussion218. 10.1111/j.1525-1403.2011.00348.x 21992242

[B36] Pashut T. Magidov D. Ben-Porat H. Wolfus S. Friedman A. Perel E. (2014). Patch-clamp recordings of rat neurons from acute brain slices of the somatosensory cortex during magnetic stimulation. *Front. Cell Neurosci.* 8:145. 10.3389/fncel.2014.00145 24917788 PMC4042461

[B37] Polikov V. S. Tresco P. A. Reichert W. M. (2005). Response of brain tissue to chronically implanted neural electrodes. *J. Neurosci. Methods* 148 1–18. 10.1016/j.jneumeth.2005.08.015 16198003

[B38] Radman T. Ramos R. L. Brumberg J. C. Bikson M. (2009). Role of cortical cell type and morphology in subthreshold and suprathreshold uniform electric field stimulation in vitro. *Brain Stimul.* 2 e211–e213. 10.1016/j.brs.2009.03.007 20161507 PMC2797131

[B39] Rahman A. Reato D. Arlotti M. Gasca F. Datta A. Parra L. C. (2013). Cellular effects of acute direct current stimulation: Somatic and synaptic terminal effects. *J. Physiol.* 591 2563–2578. 10.1113/jphysiol.2012.247171 23478132 PMC3678043

[B40] Rastogi P. Lee E. G. Hadimani R. L. Jiles D. C. (2017). Transcranial magnetic stimulation-coil design with improved focality. *AIP Adv.* 7:056705. 10.1063/1.4973604

[B41] Rattay F. (1986). Analysis of models for external stimulation of axons. *IEEE Trans. Biomed. Eng.* 33 974–977. 10.1109/TBME.1986.325670 3770787

[B42] Rattay F. (1989). Analysis of models for extracellular fiber stimulation. *IEEE Trans. Biomed. Eng.* 36 676–682. 10.1109/10.32099 2744791

[B43] Rattay F. Resatz S. (2007). Dipole distance for minimum threshold current to stimulate unmyelinated axons with microelectrodes. *IEEE Trans. Biomed. Eng.* 54 158–162. 10.1109/TBME.2006.883730 17260868

[B44] Ravazzani P. Ruohonen J. Grandori F. Tognola G. (1996). Magnetic stimulation of the nervous system: induced electric field in unbounded, semi-infinite, spherical, and cylindrical media. *Ann. Biomed. Eng.* 24 606–616. 10.1007/BF02684229 8886241

[B45] Saha R. Faramarzi S. Bloom R. P. Benally O. J. Wu K. di Girolamo A. (2022a). Strength-frequency curve for micromagnetic neurostimulation through excitatory postsynaptic potentials (EPSPs) on rat hippocampal neurons and numerical modeling of magnetic microcoil (mu coil). *J. Neural Eng.* 19. 10.1088/1741-2552/ac4baf 35030549

[B46] Saha R. Wu K. Bloom R. Liang S. Tonini D. Wang J. P. (2022b). A review on magnetic and spintronic neurostimulation: challenges and prospects. *Nanotechnology* 33. 10.1088/1361-6528/ac49be 35013010

[B47] Saito A. (2021). Fabrication of a miniature figure-of-eight coil for micromagnetic stimulation on neuronal tissue. *URSI Radio Sci. Lett.* 3. 10.46620/21-0008

[B48] Sankarasubramanian V. Buitenweg J. R. Holsheimer J. Veltink P. (2011). Electrode alignment of transverse tripoles using a percutaneous triple-lead approach in spinal cord stimulation. *J. Neural. Eng.* 8:016010. 10.1088/1741-2560/8/1/016010 21248383

[B49] Sekino M. Ohsaki H. Takiyama Y. Yamamoto K. Matsuzaki T. Yasumuro Y. (2015). Eccentric figure-eight coils for transcranial magnetic stimulation. *Bioelectromagnetics* 36 55–65. 10.1002/bem.21886 25399864

[B50] Tai C. de Groat W. C. Roppolo J. R. (2005). Simulation analysis of conduction block in unmyelinated axons induced by high-frequency biphasic electrical currents. *IEEE Trans. Biomed. Eng.* 52 1323–1332. 10.1109/tbme.2005.847561 16041996 PMC2820275

[B51] Talebinejad M. Musallam S. (2010). Effects of TMS coil geometry on stimulation specificity. *Annu. Int. Conf. IEEE Eng. Med. Biol. Soc.* 2010 1507–1510. 10.1109/IEMBS.2010.5626840 21096368

[B52] Tanner J. A. (1962). Reversible blocking of nerve conduction by alternating-current excitation. *Nature* 195 712–713. 10.1038/195712b0 13919574

[B53] Tischler H. Wolfus S. Friedman A. Perel E. Pashut T. Lavidor M. (2011). Mini-coil for magnetic stimulation in the behaving primate. *J. Neurosci. Methods* 194 242–251. 10.1016/j.jneumeth.2010.10.015 20974177

[B54] Ueno S. Sekino M. (2021). Figure-eight coils for magnetic stimulation: from focal stimulation to deep stimulation. *Front. Hum. Neurosci.* 15:805971. 10.3389/fnhum.2021.805971 34975440 PMC8716496

[B55] Ueno S. Tashiro T. Harada K. (1988). Localized stimulation of neural tissues in the brain by means of a paired configuration of time-varying magnetic fields. *J. Appl. Phys.* 64 5862–5864. 10.1063/1.342181

[B56] van den Munckhof P. Contarino M. F. Bour L. J. Speelman J. D. de Bie R. M. Schuurman P. R. (2010). Postoperative curving and upward displacement of deep brain stimulation electrodes caused by brain shift. *Neurosurgery* 67 49–53; discussion53–44. 10.1227/01.NEU.0000370597.44524.6D 20559091

[B57] Walsh V. Pascual-Leone A. (2003). *Transcranial magnetic stimulation: A neurochronometrics of mind.* Cambridge, MA: The MIT Press.

[B58] Yamamoto K. Miyawaki Y. Saitoh Y. Sekino M. (2016). Improvement in efficiency of transcranial magnetic stimulator coil by combination of iron core plates laminated in different directions. *IEEE Trans. Magn.* 52:5100504. 10.1109/TMAG.2016.2514321

[B59] Yang X. Zou H. Jung G. Richard G. Linke S. J. Ader M. (2013). Nonneuronal control of the differential distribution of myelin along retinal ganglion cell axons in the mouse. *Invest. Ophthalmol. Vis. Sci.* 54 7819–7827. 10.1167/iovs.13-12596 24222305

[B60] Ye H. (2022). Finding the location of axonal activation by a miniature magnetic coil. *Front. Comput. Neurosci.* 16:932615. 10.3389/fncom.2022.932615 35847967 PMC9276924

[B61] Ye H. Barrett L. (2021). Somatic inhibition by microscopic magnetic stimulation. *Sci. Rep.* 11:13591. 10.1038/s41598-021-93114-x 34193906 PMC8245477

[B62] Ye H. Steiger A. (2015). Neuron matters: Electric activation of neuronal tissue is dependent on the interaction between the neuron and the electric field. *J. Neuroeng. Rehabil.* 12:65. 10.1186/s12984-015-0061-1 26265444 PMC4534030

[B63] Ye H. Chen V. C. Helon J. Apostolopoulos N. (2020). Focal suppression of epileptiform activity in the hippocampus by a high-frequency magnetic field. *Neuroscience* 432 1–14. 10.1016/j.neuroscience.2020.02.018 32105740

[B64] Yu D. Y. Cringle S. J. Balaratnasingam C. Morgan W. H. Yu P. K. Su E. N. (2013). Retinal ganglion cells: Energetics, compartmentation, axonal transport, cytoskeletons and vulnerability. *Prog. Retin. Eye Res.* 36 217–246. 10.1016/j.preteyeres.2013.07.001 23891817

[B65] Zaaimi B. Ruiz-Torres R. Solla S. A. Miller L. E. (2013). Multi-electrode stimulation in somatosensory cortex increases probability of detection. *J. Neural Eng.* 10:056013. 10.1088/1741-2560/10/5/056013 23985904 PMC3821924

[B66] Zhang D. Cui X. Zheng J. Zhang S. Wang M. Lu W. (2021). Neurosurgical robot-assistant stereoelectroencephalography system: Operability and accuracy. *Brain Behav.* 11:e2347. 10.1002/brb3.2347 34520631 PMC8553331

