# Supplementary figures and images for: Improving focality and consistency in micromagnetic stimulation
Source: Front Comput Neurosci. 2023 Feb 2;17:1105505. doi: 10.3389/fncom.2023.1105505 (PMC9932264; doi:10.3389/fncom.2023.1105505)

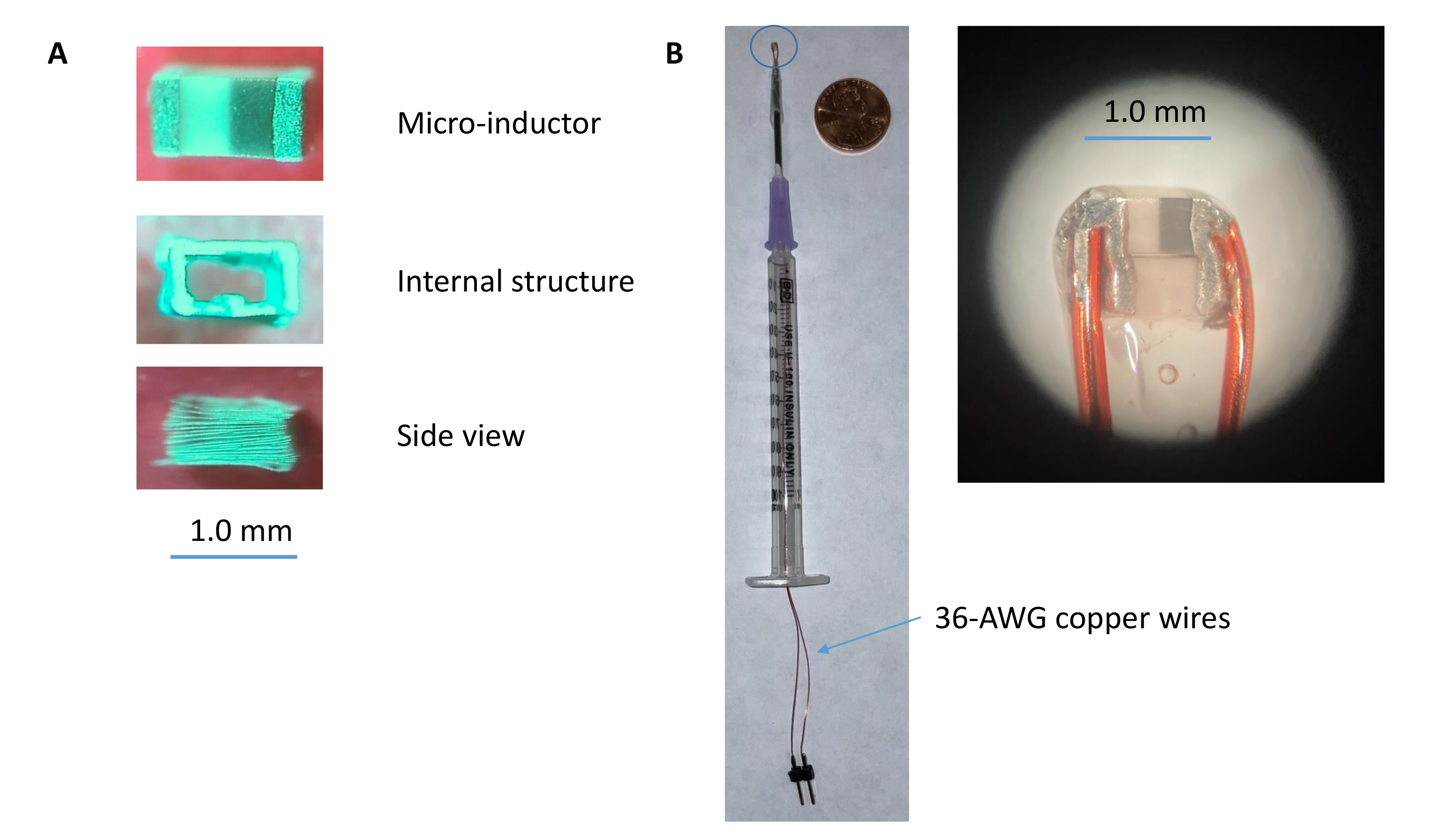

Supplement: Supplementary Figure 1 — Figure-eight micro-coil used in neural stimulation experiments. The coil was assembled from two commercially available inductors. (A) Coil internal structure revealed by the chemical removal of the encapsulation. (B) Assembly of the figure-eight coil with the two inductors stacked on top of each other, with opposite wire winding. [file Image_1.tif]

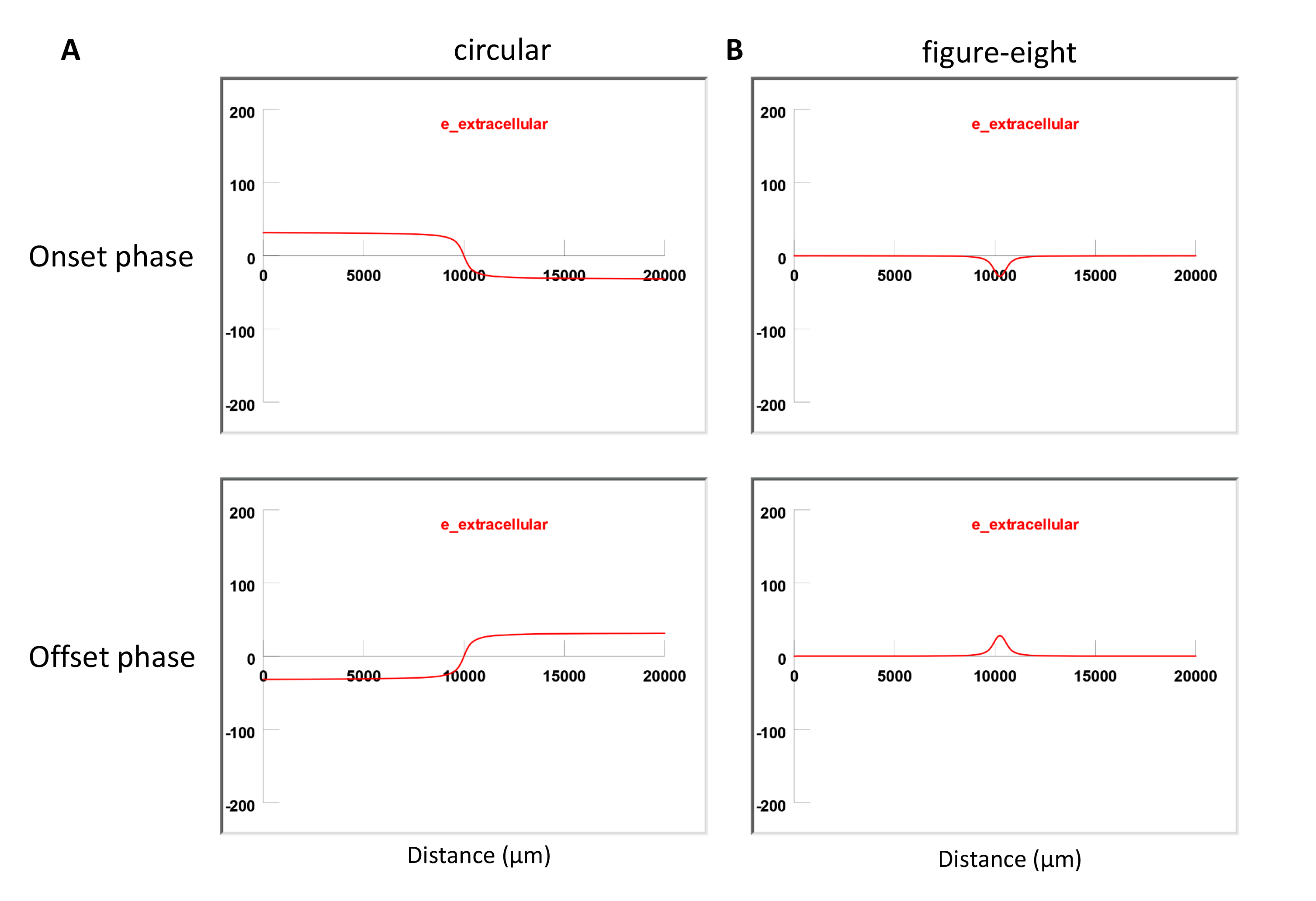

Supplement: Supplementary Figure 2 — Extracellular potential along the modeled axon in NEURON simulation. The potential was generated by a positive pulse delivered through a circular micro-coil (A) versus a figure-eight micro-coil (B). [file Image_2.tif]
